
JOURNAL INFORMATION
==============================
NLM Title Abbreviation: J Diabetes Investig
Journal ID: J Diabetes Investig
NLM Journal ID: 101520702
Journal ID: JDI

Journal of Diabetes Investigation

ISSN: 2040-1116
EISSN: 2040-1124
Publisher: Wiley

ARTICLE INFORMATION
==============================
PMCID: PMC11474793
DOI: 10.1111/jdi.13854
Article ID: JDI13854
Article version: 1
Subjects: Issue Information, Issue Information

Issue Information

Electronic publication date: 2023 Jul 21
Publication date: 2023 Aug
Volume: 14
Issue: 8
First page: 921
Last page: 922
Copyright: © 2023 The Authors. Journal of Diabetes Investigation published by Asian Association for the Study of Diabetes (AASD) and John Wiley & Sons Australia, Ltd
License: This is an open access article under the terms of the http://creativecommons.org/licenses/by/4.0/ License, which permits use, distribution and reproduction in any medium, provided the original work is properly cited.
License URL: https://creativecommons.org/licenses/by/4.0/

Figure count: 0
Table count: 0
Page count: 5

source-schema-version-number: 2.0
cover-date: August 2023
details-of-publishers-convertor: Converter:WILEY_ML3GV2_TO_JATSPMC version:6.4.9 mode:remove_FC converted:15.10.2024

==============================

### JDI

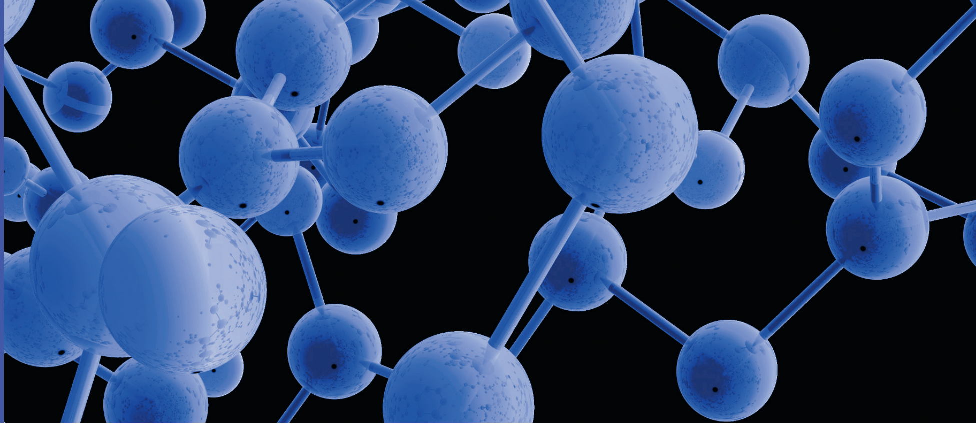

Volume 14 | Issue 8
August 2023

### Journal of Diabetes Investigation

Official Journal of the Asian Association for the Study of Diabetes

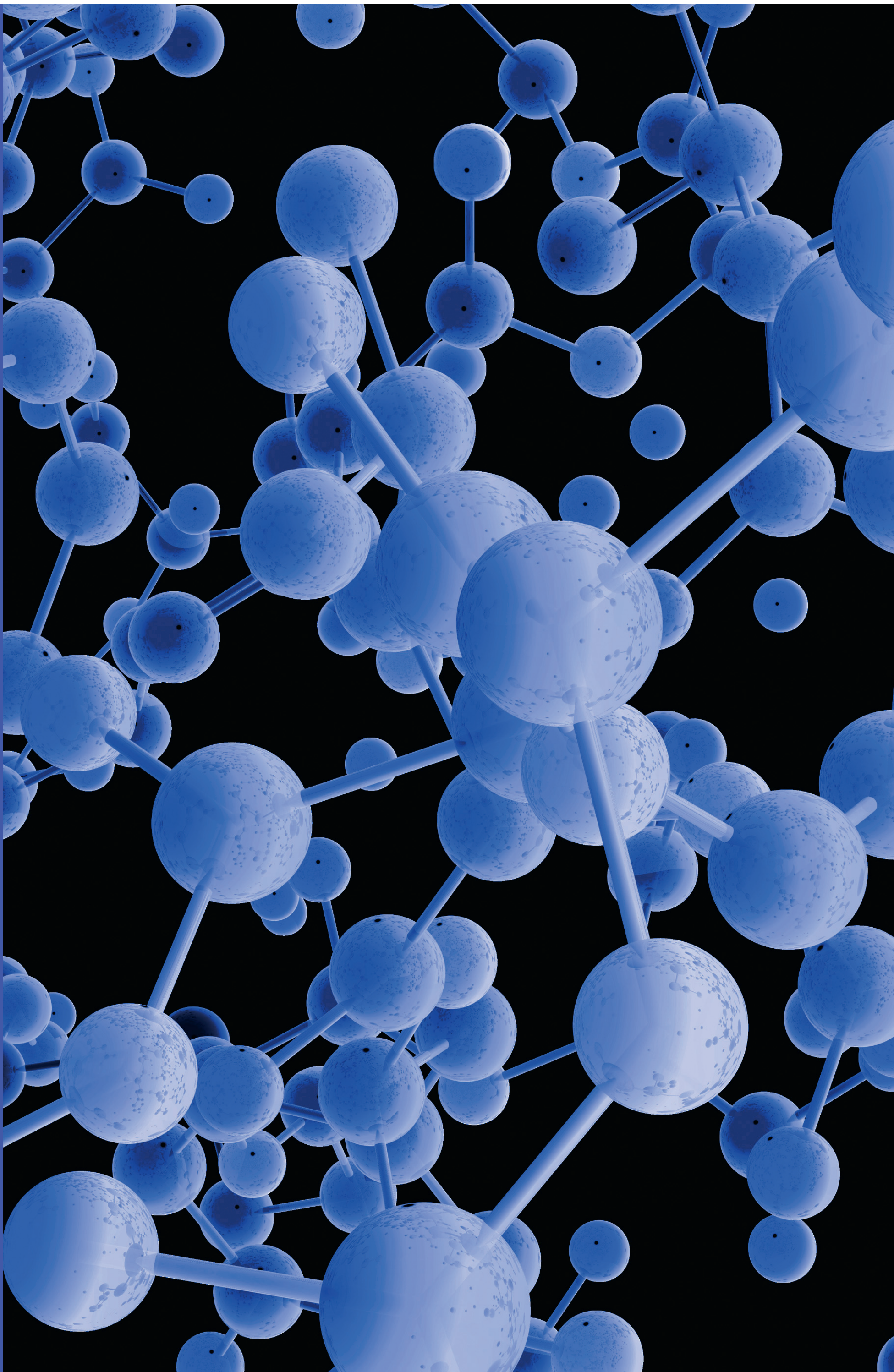

HbA1c and telemedicine during COVID-19

THE ASIAN ASSOCIATION FOR THE STUDY OF DIABETES

WILEY

JDI Journal of Diabetes Investigation

Open access

Official Journal of the Asian Association for the Study of Diabetes

**Editor-in-Chief**
***Nigishi Hotta,*** *Japan Organization of Occupational Health and Safety, Chubu Rosai Hospital, Nagoya, Japan*

**Co-Editor**
***Domenico Accili,*** *Columbia University, New York, USA*

**Expert Editor**
***Bo Ahren,*** *Lund, Sweden*
***Young Min Cho,*** *Seoul, Korea*
***Sean Dinneen,*** *Galway, Ireland*
***Marc Gilbert,*** *Montpelier, France*
***Limei Liu,*** *Shanghai, China*
***Ronald Ma,*** *Hong Kong, China*
***Louis Philipson,*** *Chicago, USA*
***Wayne H-H Sheu,*** *Taichung, Taiwan*
***Bernard Thorens,*** *Lausanne, Switzerland*
***Daisuke Yabe,*** *Gifu, Japan*

**Editorial Board**
***Eiichi Araki,*** *Kumamoto, Japan*
***Xiaoping Chen,*** *Beijing, China*
***Lee-Ming Chuang,*** *Taipei, Taiwan*
***Masakazu Haneda,*** *Asahikawa, Japan*
***Larry Low-Tone Ho,*** *Taipei, Taiwan*
***Tianpei Hong,*** *Beijing, China*
***Akihisa Imagawa,*** *Osaka, Japan*
***Nobuya Inagaki,*** *Kyoto, Japan*
***Linong Ji,*** *Beijing, China*
***Weiping Jia,*** *Shanghai, China*
***Yi-Der Jiang,*** *Taipei, Taiwan*
***Takashi Kadowaki,*** *Tokyo, Japan*
***Kohei Kaku,*** *Kurashiki, Japan*
***Keizo Kanasaki,*** *Izumo, Japan*
***Doo-Man Kim,*** *Seoul, Korea*
***Karen Lam,*** *Hong Kong, China*
***Hong Kyu Lee,*** *Gyeonggi-do, Korea*
***In-Kyu Lee,*** *Daegu, Korea*
***Moon-Kyu Lee,*** *Seoul, Korea*
***Jiro Nakamura,*** *Nagakute, Japan*
***Kishio Nanjo,*** *Wakayama, Japan*
***Wataru Ogawa,*** *Kobe, Japan*
***Yutaka Seino,*** *Osaka, Japan*
***Shyi-Jang Shin,*** *Kaohsiung, Taiwan*
***Hirohito Sone,*** *Niigata, Japan*
***Kathryn Tan,*** *Hong Kong, China*
***Yukio Tanizawa,*** *Ube, Japan*
***Kohjiro Ueki,*** *Tokyo, Japan*
***Jun Wada,*** *Okayama, Japan*
***Hirotaka Watada,*** *Tokyo, Japan*
***Jianping Weng,*** *Guangzhou, China*
***Soroku Yagihashi,*** *Hirosaki, Japan*
***Hiroshi Yamamoto,*** *Kanazawa, Japan*
***Toshimasa Yamauchi,*** *Tokyo, Japan*
***Wenying Yang,*** *Beijing, China*
***Kazuki Yasuda,*** *Tokyo, Japan*
***Koutaro Yokote,*** *Chiba, Japan*
***Kun-Ho Yoon,*** *Seoul, Korea*
***Dalong Zhu,*** *Nanjing, China*

**International Editorial Board**
***Michael A. Brownlee,*** *New York, USA*
***Subrata Chakrabarti,*** *Ontario, Canada*
***Mark E. Cooper,*** *Prahran, Australia*
***Philippe Froguel,*** *London, UK*
***Wilfred Fujimoto,*** *Hawaii, USA*
***Michael S. German,*** *San Francisco, USA*
***Andrew Hattersley,*** *Exeter, UK*
***Jean-Claude Henquin,*** *Brussels, Belgium*
***Jens Juul Holst,*** *Copenhagen, Denmark*
***Frank B. Hu,*** *Boston, USA*
***C. Ronald Kahn,*** *Boston, USA*
***George L. King,*** *Boston, USA*
***Rayaz A. Malik,*** *Manchester, UK*
***Michael Mauer,*** *Minneapolis, USA*
***Carl Erik Mogensen,*** *Aarhus, Denmark*
***Steven E. Shoelson,*** *Boston, USA*
***Jay S. Skyler,*** *Miami, USA*
***Paul Zimmet,*** *Melbourne, Australia*

**Assistant Editorial Board**
***Tien-Jyun Chang,*** *Taipei, Taiwan*
***Yoshiyuki Hamamoto,*** *Osaka, Japan*
***Norio Harada,*** *Kyoto, Japan*
***Yi-Jen Hung,*** *Taipei, Taiwan*
***Chi-Min Hwu,*** *Taipei, Taiwan*
***Hideki Kamiya,*** *Aichi, Japan*
***Dae Jung Kim,*** *Suwon, Korea*
***Seung-Hwan Lee,*** *Seoul, Korea*
***Hung-Yuan Li,*** *Taipei, Taiwan*
***Takashi Matsuzaka,*** *Tsukuba, Japan*
***Keiko Naruse,*** *Aichi, Japan*
***Takeshi Nishikawa,*** *Kumamoto, Japan*
***Eung-Jung Rhee,*** *Seoul, Korea*
***Yusuke Seino,*** *Aichi, Japan*
***Hironori Waki,*** *Tokyo, Japan*

**Aims and Scope:** *Journal of Diabetes Investigation* is your core diabetes journal from Asia; the official journal of the Asian Association for the Study of Diabetes (AASD). The journal publishes original research, country reports, commentaries, reviews, mini-reviews, case reports, letters, as well as editorials and news. The journal embraces clinical and experimental research in diabetes and related areas. This includes aspects of prevention, treatment, as well as molecular aspects and pathophysiology. Translational research focused on the exchange of ideas between clinicians and researchers is also welcome.

**Address for Editorial Correspondence:**
The Editor, *Journal of Diabetes Investigation*, Nomura Fudosan Nishi Shinjuku Building, 8F 8-4-2 Nishi Shinjuku, Shinjuku-ku, Tokyo 160-0023, Japan. Tel: +81 3 4520 9009; Fax: +81 3 4520-9059 Email: JDI@wiley.com

**Copyright © 2023 Asian Association for the Study of Diabetes and John Wiley & Sons Australia, Ltd**

**Cover:** Insulin molecule © Pasieka/Science Photo Library

This journal is available online at Wiley Online Library. Visit wileyonlinelibrary.com to search the articles and register for table of contents and e-mail alerts.

**For submission instructions, and all other information visit wileyonlinelibrary.com/journal/jdi**

ISSN 2040-1124 (Online)

JDI.JEB.Aug23

WILEY

**Publisher**
*Journal of Diabetes Investigation* is published by John Wiley & Sons Australia, Ltd
Level 19, 15 William Street,
Melbourne, Victoria 3000,
Australia.
Tel: +61 (0)3 9274 3100

**Production Editor**
Jayalakshmi Mohan (email: JDIproduction@wiley.com)

**Abstracting and Indexing Services**
The Journal is indexed by Academic OneFile (GALE Cengage), Biological Abstracts (Clarivate Analytics), Biological Science Database (ProQuest), BIOSIS Previews (Clarivate Analytics), Embase (Elsevier), Health & Medical Collection (ProQuest), Health Research Premium Collection (ProQuest), Hospital Premium Collection (ProQuest), MEDLINE/PubMed (NLM), Natural Science Collection (ProQuest), ProQuest Central (ProQuest), Science Citation Index Expanded (Clarivate Analytics), SciTech Premium Collection (ProQuest), SCOPUS (Elsevier), Web of Science (Clarivate Analytics).

**Open Access and Copyright**
All articles published by *Journal of Diabetes Investigation* are fully open access: immediately freely available to read, download and share. All *Journal of Diabetes Investigation* articles accepted from 24 September 2013 are published under the terms of the Creative Commons Attribution 4.0 Unported (CC BY 4.0) license, which permits use, distribution and reproduction in any medium, provided the original work is properly cited. Articles accepted before this date were published under the agreement as stated in the final article. Copyright on any *Journal of Diabetes Investigation* article accepted after 24 September 2013, the date on which *Journal of Diabetes Investigation* became an open access journal, is retained by the author(s). Authors grant to *Journal of Diabetes Investigation* and Wiley a license to publish the article and for Wiley to identify itself as the original publisher. Authors also grant any third party the right to use the article freely as long as its integrity is maintained and its original authors, citation details and publisher are identified. Please visit https://authorservices.wiley.com/author-resources/Journal-Authors/open-access/hybrid-open-access.html for further information about Open Access.

**Use by commercial "for-profit" organisations (not applicable to CC BY 4.0 licensed articles)**
Use of Wiley Open Access articles for commercial, promotional, or marketing purposes requires further explicit permission from Wiley and will be subject to a fee. Requests to reproduce material from this journal content are being handled through the Rightslink® online service.

- Locate the article you wish to reproduce on Wiley Online Library (http://onlinelibrary.wiley.com)
- Navigate to the abstract page
- Click on the 'Request Permissions' link, under the 'ARTICLE TOOLS' menu
- Follow the online instructions and select your requirements from the drop down options and click on 'quick price' to get a quote
- Create a RightsLink® account to complete your transaction (and pay, where applicable)
- Read and accept our Terms & Conditions and Download your license
- For any technical queries please contact customercare@copyright.com
- For further information and to view a Rightslink® demo please visit www.wiley.com and select Rights & Permissions.
- Print reprints of Wiley Open Access articles can be purchased from corporatesales@wiley.com.

**Disclaimer**
The Publisher, the Asian Association for the Study of Diabetes, and Editors cannot be held responsible for errors or any consequences arising from the use of information contained in this journal; the views and opinions expressed do not necessarily reflect those of the Publisher, the Asian Association for the Study of Diabetes, or Editors, neither does the publication of advertisements constitute any endorsement by the Publisher, the Asian Association for the Study of Diabetes, or Editors of the products advertised.

Wiley Open Access articles posted to repositories or websites are without warranty from Wiley of any kind, either express or implied, including, but not limited to, warranties of merchantability, fitness for a particular purpose, or noninfringement.
To the fullest extent permitted by law Wiley disclaims all liability for any loss or damage arising out of, or in connection, with the use of or inability to use the content.

**Journal Customer Services:** For ordering information, claims and any enquiry concerning your journal subscription please go to https://wolsupport.wiley.com/s/contactsupport?tabset-a7d10=2 or contact your nearest office.
**Americas:** Email: cs-journals@wiley.com; Tel: +1 877 762 2974
**Europe, Middle East and Africa:** Email: cs-journals@wiley.com; Tel: +44 (0) 1865 778315; 0800 1800 536 (Germany)
**Germany, Austria, Switzerland, Luxembourg, Liechtenstein:** cs-germany@wiley.com; Tel: 0800 1800 536 (Germany)
**Asia Pacific:** Email: cs-journals@wiley.com; Tel: +65 3165 0890.
**Japan:** For Japanese speaking support, Email: cs-japan@wiley.com; .
**Visit our Online Customer Help** at https://wolsupport.wiley.com/s/contactsupport?tabset-a7d10=2

Wiley's Corporate Citizenship initiative seeks to address the environmental, social, economic, and ethical challenges faced in our business and which are important to our diverse stakeholder groups. Since launching the initiative, we have focused on sharing our content with those in need, enhancing community philanthropy, reducing our carbon impact, creating global guidelines and best practices for paper use, establishing a vendor code of ethics, and engaging our colleagues and other stakeholders in our efforts. Follow our progress at www.wiley.com/go/citizenship

VOLUME 14 – ISSUE 8 – AUGUST 2023

### Journal of Diabetes Investigation

Open access

### Mini Review

**Delivering value-based healthcare for people with diabetes in a national publicly funded health service: Lessons from Ireland and Wales**
Máire T O'Donnell, Sally Lewis, Sarah Davies, and Sean F Dinneen **925**

### Commentaries

**Is a low-carbohydrate, high-fat diet feasible for people with type 2 diabetes and nonalcoholic fatty liver disease?**
Fu-Shun Yen, Pei-Chuan Lee, and Chii-Min Hwu **930**

**Is caloric restriction enough to increase longevity? Fasting and circadian alignment**
Yoshiyuki Hamamoto, Takeshi Kurose, and Yutaka Seino **933**

### Update

**Diet and exercise are a fundamental part of comprehensive care for type 2 diabetes**
Yun Kai Yeh, Fu-Shun Yen, and Chii-Min Hwu **936**

### Original Articles

### Basic Science and Research

**Long non-coding ribonucleic acid zinc finger E-box binding homeobox 1 antisense RNA 1 regulates myocardial fibrosis in diabetes through the Hippo–Yes-associated protein signaling pathway**
Jing Wu, Rui Lyu, Shumin Chen, and Xiaoguang Wang **940**

### Clinical Science and Care

**Association of the triglyceride-glucose index with subclinical left ventricular dysfunction in type 2 diabetes mellitus patients: A retrospective cross-sectional study**
Qi-chao Sun, Jie Liu, Ran Meng, Ning Zhang, Jing Yao, Fan Yang, and Da-long Zhu **953**

**Predictive values of metabolic score for insulin resistance on risk of major adverse cardiovascular events and comparison with other insulin resistance indices among Chinese with and without diabetes mellitus: Results from the 4C cohort study**
Limeng Pan, Huajie Zou, Xiaoyu Meng, Danpei Li, Wenjun Li, Xi Chen, Yan Yang, and Xuefeng Yu **961**

**Maternal uric acid levels and risk of gestational diabetes mellitus: A systematic review and dose–response meta-analysis of cohort studies including 105,380 participants**
Ali Nikparast, Jamal Rahmani, Reza Bagheri, Saba Mohammadpour, Mehdi Shadnoosh, Alexei Wong, and Matin Ghanavati **973**

**Impact of the COVID -19 pandemic on the glycemic control in people with diabetes mellitus: A retrospective cohort study**
Kanako Ohkuma, Mika Sawada, Masakazu Aihara, Shunsuke Doi, Rie Sekine, Satoshi Usami, Kazuhiko Ohe, Naoto Kubota, and Toshimasa Yamauchi **985**

**Impact of COVID -19 pandemic on behavioral changes and glycemic control and a survey of telemedicine in patients with diabetes: A multicenter retrospective observational study**
Ryotaro Bouchi, Takehiro Sugiyama, Atsushi Goto, Mitsuru Ohsugi, Narihito Yoshioka, Hideki Katagiri, Tomoya Mita, Yushi Hirota, Hiroshi Ikegami, Munehide Matsuhisa, Eiichi Araki, Hiroki Yokoyama, Masae Minami, Katsuya Yamazaki, Hideaki Jinnouchi, Hiroki Ikeda, Hitomi Fujii, Miyuki Nogawa, Masahiro Kaneshige, Kengo Miyo, and Kohjiro Ueki **994**

### Case Report

**A case of fulminant type 1 diabetes and protein C deficiency complicated by deep vein thrombosis**
Masato Kohata, Shinjiro Kodama, Nobuhiro Yaoita, Shinichiro Hosaka, Kei Takahashi, Keizo Kaneko, Junta Imai, Satoshi Yasuda, and Hideki Katagiri **1005**

### Letter to the Editor

**Fibroblast growth factor 21 as a candidate of a novel serum biomarker for mitochondrial diabetes**
Yuji Komorita, Akiyo Shiroozu, and Hidetoshi Nakamura **1009**
